# The relationship between corneal biomechanical parameters and treatment outcomes of orthokeratology lenses

**DOI:** 10.1186/s12886-022-02480-1

**Published:** 2022-06-11

**Authors:** Xia Li, Jianjiang Xu, Jiaxu Hong, Jing Yao

**Affiliations:** 1 Department of Ophthalmology, Shanghai Aier Eye Hospital, Shanghai, China; 2 Department of Ophthalmology and Visual Science, Shanghai Medical College, Eye, Ear, Nose, and Throat Hospital, Fudan University, No. 83 Fenyang Road, Xuhui District, Shanghai, 200031 China; 3 Key Laboratory of Myopia of State Health Ministry and Key Laboratory of Visual Impairment and Restoration of Shanghai, Eye, Ear, Nose and Throat Hospital, Fudan University, Shanghai, China

**Keywords:** Ambrósio’s relational thickness to the horizontal profile, Orthokeratology lenses, Myopia progression, Corneal biomechanical properties

## Abstract

**Background:**

To evaluate changes in corneal biomechanical properties after long-term orthokeratology (OK) treatment and the factors affecting treatment outcomes.

**Methods:**

Twenty-four myopic teenagers who wore OK lenses for more than 1 year were included. Twenty-three individuals of the same age and with the same spherical equivalent wearing single-vision spectacles (SVS) were enrolled as controls. After routine eye examinations, corneal biomechanical properties and axial length were measured. Parameters were compared between groups.

**Results:**

Less axial elongation (AE) occurred in the OK group (*P* = 0.021). The OK group experienced a statistically significant decrease in the A1 deformation amplitude (*P* = 0.02), whole eye movement maximum (*P* = 0.026), and Ambrósio’s relational thickness to the horizontal profile (ARTh) (*P* < 0.001), and a statistically significant increase in the pachyslope (*P* < 0.001) and Corvis biomechanical index (*P* < 0.001). Smaller ARTh and a larger highest concavity deflection area resulted in a better refractive state. The inhibitory effect of AE was better for older patients with smaller ARTh.

**Conclusions:**

Long-term OK treatment slowed myopia progression by reshaping the cornea. Smaller ARTh after OK lens wear indicated a better refractive state and slower AE and could predict OK lens treatment outcomes.

## Background

The worldwide prevalence of myopia is high for adolescents. With myopic progression, the risk of vision-threatening ocular complications greatly increases [1–4]. To slow the progression of myopia, a number of pharmacological and optical treatment strategies have been introduced. Orthokeratology (OK) is an effective clinical modality that uses specially designed rigid contact lenses to temporarily reduce myopia [5–9]. It also has an inhibitory effect on axial elongation (AE) in adolescents with myopia. Compared to single-vision spectacles (SVS), OK lenses have been shown to effectively slow AE by 36% to 63% [8, 10–15].

The treatment effects of OK lenses are achieved mainly by flattening the central cornea [5, 7]. Previous studies of short-term OK treatment reported significant flattening of anterior corneal curvatures and decreases in central corneal thickness (CCT) even after as little as 10 min, thereby reducing the spherical equivalent (SE) refraction and improving uncorrected visual acuity [16–18]. Lu et al [19] examined the change in the ocular surface after 15 min and proposed that corneal malleability may be a predictor of OK treatment outcomes.

Because the cornea is a viscoelastic tissue, Alharbi [20] hypothesized that the positive pressure generated by the center of the reverse geometry lenses may alter the corneal biomechanics, which may have a role in the effects of OK treatment. Both the ORA and Corvis ST measure corneal deformation in response to an air puff. The first device developed to assess corneal biomechanical properties *in vivo* is the Ocular Response Analyzer (ORA). The ORA measures corneal behavior during a bidirectional applanation process induced by an air jet and estimates corneal hysteresis (CH) and the corneal resistance factor (CRF), along with a set of 38 waveform-derived parameters. The CRF has been shown to decrease with the increasing duration of wear after short-term OK treatment (15, 30, and 60 min and overnight) [21]. González-Méijome [22] observed a faster response and recovery with OK treatment with lower CH. After 30 nights of OK treatment, individuals experienced a 2.5% decrease in CH and 7.1% decrease in CRF, suggesting that the cornea developed the tendency to rebound slightly faster and became less resistant to an applied force after treatment [23]. However, all these studies focused on short-term OK treatment. To our knowledge, no study has monitored the corneal biomechanical properties after more than 30 nights of OK treatment. According to the results of a cross-sectional study [24], a lower CH is associated with a longer axial length. A study by Wan et al [25] suggested that a lower CH and CRF may be risk factors for AE.

In clinical practice, however, not all the patients can achieve satisfactory outcomes even with sufficient OK treatment. Whether corneal biomechanical properties are related to OK treatment outcomes, especially the inhibitory effect on AE, is unknown. Elucidating the objective factors that influence treatment outcomes will provide guidance for the management of these patients. Therefore, the aim of the current study was to investigate the changes in the corneal biomechanical properties after OK treatment for 1 year using Corvis ST (Oculus, Wetzlar, Germany), which is a device that can be used to assess corneal biomechanical properties. Corvis ST is equipped with an ultra-high-speed Scheimpflug camera to capture images of corneal deformation at a rate of 4,330 frames per second for approximately 30 ms, thereby enabling a direct assessment of the deformation response [26]. In this cross-sectional study, we used Corvis ST to evaluate the corneal mechanical properties after long-term OK treatment. We also aimed to determine the relationship between biomechanical properties and OK treatment outcomes.

## Methods

This study followed the tenets of the Declaration of Helsinki and was approved by the ethics committee of the Eye, Ear, Nose, and Throat Hospital of Fudan University. Written informed consent, including consent to allow the use of clinical data for scientific purposes, was obtained from all participants.

### Subjects

This study included 46 eyes of 24 individuals who wore OK lenses for more than 1 year (OK group). An additional 46 eyes of 23 individuals of the same age and with the same SE who wore regular SVS were included as controls (SVS group). The best-corrected visual acuity was at least 0.10 logMAR for each eye. Individuals with a history of contact lens use or cessation of lens use for more than 30 days were excluded. Individuals with any ocular disease except refractive error, individuals who had undergone ocular surgery, individuals who had any general disease that may affect corneal biomechanical properties (e.g., connective tissue disorders), and individuals who were using topical or systemic medications were also excluded.

### OK lenses

The OK lenses worn by the participants were spherical, four-zone, reverse geometry, gas-permeable, rigid contact lenses (Emerald Series; Euclid, Sterling, VA) composed of oprifocon A (Boston Equalens II; Bausch and Lomb, Rochester, NY). All lenses were prescribed by an experienced optometrist. Those whose myopic degree was more than -5 D received the -5 D OK lens, and the residual myopic degree was corrected using SVS during the day. The visual acuity, refractive state, slit-lamp examination results of the anterior segment, and the integrity of the OK lenses were observed every 3 months to confirm the efficacy and safety of the treatment. All examinations were conducted 4 h after the OK lenses were removed during the morning.

### Measurements of corneal biomechanical properties

Corvis ST (software version 1.3r1469) uses a precisely metered air pulse to deform the cornea and an ultra-high-speed camera that utilizes Scheimpflug geometry to capture images of the horizontal meridian at more than 4,300 frames per second, resulting in 140 images during a 30-ms air puff [26]. The output dynamic corneal response (DCR) parameters of Corvis ST include the Corvis biomechanical idex (CBI), stiffness parameter at first applanation (SP-A1), stiffness parameter at highest concavity (SP-HC), intraocular pressure (IOP), biomechanically corrected IOP (bIOP), CCT, Ambrósio’s relational thickness to the horizontal profile (ARTh), PachySlope, first applanation (A1) parameters (A1 time, A1 length, A1 velocity, A1 deformation amplitude, and A1 deflection amplitude), second applanation (A2) parameters (A2 time, A2 length, A2 velocity, A2 deformation amplitude, and A2 deflection amplitude), highest concavity (HC) parameters (HC time, HC radius, HC deformation amplitude, HC deflection amplitude, HC deflection area), deformation amplitude maximum, deflection amplitude maximum, peak distance, whole eye movement maximum, and others [27].

### Other clinical assessments

Visual acuity was measured using the tumbling E Early Treatment Diabetic Retinopathy Study charts and converted to the logarithm of the minimum angle of resolution (logMAR) scale. The cycloplegic refractive status was determined using a Nidek 9000 autorefractor (Nidek Co., Ltd., Gamagori, Aichi, Japan). Corneal topography was measured using Pentacam (Oculus, Wetzlar, Germany). The axial length was measured using IOLMaster (Carl Zeiss Jena GmbH, Jena, Germany). The average 12-month axial length change was calculated by subtracting the baseline axial length from the most recent value, dividing by the total months of OK wear, and then multiplying the result by 12.

### Statistical analysis

IBM SPSS Statistics 20.0 (IBM Corp., Armonk, NY) was used to perform the statistical analysis. The Kolmogorov–Smirnov test was applied to all data samples to check normality. Data that followed a normal distribution are presented as mean ± standard deviation (SD). Data that did not follow a normal distribution are presented as the median (lower quartile and upper quartile). Independent samples t tests were performed to determine the age difference between the OK group and SVS group. Paired *t* tests were performed to determine the differences in corneal curvature changes. Data of both eyes of the same individual were pooled in a cluster-correlated data analysis (generalized estimating equation) [28]. Significantly correlated variables were introduced in a multiple linear regression, and the stepwise method was used to develop linear regression models. Bivariate correlations were evaluated using Pearson’s correlation coefficients. A one-way analysis of variance (ANOVA) using Sigmastat 3.5 was performed to compare the data with those of previous studies (mean, SD, and sample size). Statistical significance was accepted at *P* < 0.05.

## Results

### Study participants

Ninety-two eyes of 47 individuals were included in this study: 46 eyes (50%) wore OK lenses and the other 46 eyes (50%) wore regular SVS. Thirteen eyes of nine participants had a myopic diopter greater than -5.00 D. For these patients, the myopic correction effect was demonstrated as the manifest refraction at the time of examination minus the manifest refraction at the time of OK lens fitting for -5 D. The two groups had similar demographic and clinical parameters (Table 1). None of the participants experienced adverse responses related to lens wear. No detectable abnormalities of the eyes were observed under slit-lamp microscopy.

**Table 1:** The difference of demographic features, biomechanical parameters, and clinical parameters between the OK group and the SVS group

	OK group	SVS group	*P*
Female, No. (%)	13 (54.2)	13 (56.5)	0.771^▲^
Age, year	13.1±1.8	12.3±2.6	0.254*
Spherical equivalent, D	-4.97±0.25	-4.49±0.29	0.212^■^
Axial length, mm	25.71±0.14	25.48±0.12	0.201^■^
IOP, mmHg	16.8±0.6	16.4±0.4	0.498^■^
CCT, um	530.6±7.0	538.8±5.6	0.364^■^
A1 deformation amplitude, mm	0.12±0.01	0.13±0.02	0.02^■^
Whole eye movement maximum, ms	21.65±0.24	22.29±0.14	0.026^■^
PachySlope, μm	72.59±2.15	52.34±1.67	<0.001^■^
ARTh	284.07±13.94	422.14±16.65	<0.001^■^
CBI	0.43±0.07	0.11±0.04	<0.001^■^
Axial elongation, mm (all the patients)	0.19±0.12	0.48±0.30	<0.001^■^
Axial elongation, mm (< -5.00D, n = 13)	0.23±0.04	0.55±0.06	< 0.001^■^
Axial elongation, mm (≥ -5.00D, n = 33)	0.10±0.03	0.40±0.06	<0.001^■^

*OK* orthokeratology; *SVS* single-vision spectacles; *D* diopter; *IOP* intraocular pressure; *CCT* central corneal thickness; *A1* first applanation; *ARTh* Ambrósio’s relational thickness to the horizontal profile; *CBI* Corvis biomechanical index

* Independent-samples *t* test

^▲^Chi-square test

^■^Generalized estimating equation

### Changes in topographic parameters and corneal biomechanical parameters

After OK treatment, the steep curvature and flat curvature decreased (43.4 ± 1.4 D versus 42.2 ± 0.5 D, *P*_*1*_ = 0.01; 42.3 ± 0.8 D versus 40.9 ± 0.8 D, *P*_*2*_ < 0.001). To determine whether OK treatment could alter corneal biomechanical parameters, we compared the DCR parameters of the OK group and SVS group. A1 deformation amplitude, whole eye movement maximum, and ARTh decreased more in the OK group than in the SVS group, whereas the PachySlope and CBI increased more in the OK group than in the SVS group (Table 1).

### Myopia correction after OK treatment and correlated factors

In the OK group, the manifest refraction was -0.64 ± 0.64 D at 4 h after the OK lenses were removed. The distant uncorrected visual acuity was -0.1 ± 0.2 logMAR and the distant best-corrected visual acuity was 0 ± 1.3 logMAR. The correlated factors associated with the manifest refraction included ARTh, HC deflection area, IOP, CCT, deformation amplitude maximum, A1 time, peak distance, HC deformation amplitude, HC deflection amplitude, deflection amplitude maximum, and bIOP according to Pearson’s correlation test (Table 2). Using the multiple linear regression test, ARTh and HC deflection area resulted in a better refractive state after OK treatment (Table 2).

**Table 2:** The correlated factors affecting the manifest refraction after OK treatment

Variables	Bivariate correlations*		Multiple linear regression^∆^	
	correlation coefficients	*P*	coefficients	*P*
ARTh	-.588	.002	-0.005	0.003
HC Deflection Area, mm^2^	.587	.002	0.287	0.002
IOP, mmHg	-.614	.001		
CCT, μm	-.558	.004		
Deformation amplitude maximum, mm	.617	.001		
A1-time, ms	-.581	.002		
Peak distance, mm	.431	.035		
HC deformation amplitude, mm	.617	.001		
HC deflection amplitude, mm	.485	.014		
Deflection amplitude max, mm	.439	.028		
bIOP, mmHg	-.516	.008		

*OK* Orthokeratology; *ARTh* Ambrósio’s relational thickness to the horizontal profile; *HC* Highest concavity; *IOP* Intraocular pressure; *CCT* Central corneal thickness; *A1- time* Time from the initiation of air puff until the first applanation; *bIOP* Biomechanically corrected intraocular pressure

^∆^the regression line:

manifest refraction = -0.02 + 0.285 * HC Deflection Area - 0.005 * ARTh

*Pearson’s correlation

### Inhibition of AE after OK treatment and correlated factors

The AE demonstrated by the OK group was slower than that demonstrated by the SVS group after 1 year of treatment (Table 1). OK treatment inhibited the AE by approximately 50% for 1 year. The factors associated with the growth of the axial length included ARTh, whole eye movement maximum, age, PachySlope, and CBI according to Pearson’s correlation test (Table 3). After OK treatment, the multiple linear regression test showed that older patients with smaller ARTh experienced slower growth of the axial length (Table 3).

**Table 3:** The correlated factors inhibiting axial elongation after OK treatment

Variables	Bivariate correlations*		Multiple linear regression^∆^	
	correlation coefficients	*P*	coefficients	*P*
Age, year	-0.440	<0.001	-0.041	0.001
ARTh	0.452	<0.001	0.001	0.001
whole eye movement maximum, ms	0.238	0.031		
PachySlope, um	-0.412	<0.001		
CBI	-0.331	0.002		

*OK* Orthokeratology; *ARTh* Ambrósio’s relational thickness to the horizontal profile; *CBI* Corvis Biomechanical Index.

^∆^the regression line: the growth of axial length = -0.42 + 0.001 * ARTh - 0.041 * age

*Pearson’s correlation

### OK treatment effects on higher myopia

The AE progressed faster in the higher myopia group (<-5 D) than in the lower myopia group (≥-5 D) (Table 4). For patients with higher myopia, the AE was negatively correlated with age (*r*_*1*_ = -0.412; *P*_*1*_ = 0.008), A1 dArc length (*r*_*2*_ = -0.338; *P*_*2*_ = 0.033), pachyslope (*r*_*3*_ = -0.572; *P*_*3*_ < 0.001), and CBI (*r*_*4*_ = -0.464; *P*_*4*_ = 0.003) and positively correlated with A2 deformation amplitude (*r*_*1*_= 0.329; *P*_*1*_= 0.038), A1 deflection amplitude (*r*_*2*_= 0.370; *P*_*2*_ = 0.019), A2 deflection amplitude (*r*_*3*_ = 0.312; *P*_*3*_= 0.05), and ARTh (*r*_*4*_ = 0.647; *P*_*4*_ < 0.001) according to the Pearson correlation coefficients test. All these significant correlation factors were included in the multiple linear regression, which indicated that only age (correlation coefficient = -0.205; *P* = 0.016) and ARTh (correlation coefficient = 0.002; *P* < 0.001) were correlated factors. For patients with lower myopia, the AE was negatively correlated with age (*r*_*1*_ = -0.392; *P*_*1*_ = 0.004) and pachyslope (*r*_*2*_ = -0.323; *P*_*2*_ = 0.019) and positively correlated with A1 deflection length (*r*_*1*_ = 0.348; *P*_*1*_ = 0.024) and ARTh (*r*_*2*_ = 0.328; *P*_*2*_ = 0.017) according to the Pearson correlation coefficients test. All these significant correlated factors were included in the multiple linear regression, which indicated that only age (correlation coefficient = -0.054; *P* = 0.013) and ARTh (correlation coefficient = 0.001; *P* = 0.038) were correlated factors.

**Table 4:** The difference of myopic correction and inhibitory effect of axial elongation between the two myopic sub-group

	Higher myopia group (< -5D, *n* = 13)	Lower myopia group (≥ -5D, *n* = 33)	*P*
Age, years	12.7±0.6	13.4±0.4	0.338*
Baseline myopic diopter, D	-6.08±0.13	-4.45±0.31	<0.001^■^
Residual SE, D	-0.71±0.27	-0.47±0.17	0.442^■^
Baseline axial length, mm	25.97±0.20	25.58±0.16	0.078^■^
Axial elongation, mm	0.23±0.04	0.10±0.04	0.011^■^
Baseline steep K	43.4±0.6	44.2±0.4	0.339^■^
∆ steep K	0.4±0.1	1.9±0.4	0.001^■^
Baseline flat K	42.3±0.7	42.9±0.3	0.420^■^
∆ flat K	1.0±0.4	1.6±0.1	0.140^■^

*SE* Spherical equivalent

* Independent-samples t test

^■^Generalized estimating equation

## Discussion

During this cross-sectional study, we found that OK treatment can effectively slow myopia progression. Compared to SVS treatment, OK treatment can decrease the AE by approximately 50% within 1 year. Several DCR parameters differed between these two groups. Among these factors, smaller ARTh after OK treatment was associated with a better refractive state and slower AE, suggesting that ARTh has the potential to be used as an indicator of OK lens treatment outcomes.

Overnight myopic OK lenses are designed with a reverse geometry back surface that reshapes the cornea to reduce the corneal power of refraction. This effect remains for at least 8 h to 10 h after removal of the lenses; therefore, they can correct myopia during the day. This therapeutic effect occurs because the lens base arc curvature is smaller than the corneal optical zone, which compresses the central area of the cornea mechanically and forces the corneal tissue to move from the central area to the surrounding area [29]. Eyes treated for 30 nights with OK lenses showed a thinned central corneal epithelium and a thickened, but less stratified, paracentral corneal epithelium compared with eyes treated with rigid gas-permeable (RGP) lenses for 30 nights. This probably occurred because of the redistribution of the corneal epithelium during lens wear [30]. Consistent with these results, the steep K and flat K decreased after OK treatment during our study. However, the difference in the CCT of the OK group and that of the SVS group was not statistically significant, possibly because of the low sensitivity of CCT and the longer OK treatment period during this study.

Previous studies showed that short-term and long-term OK treatment can correct myopia by flattening the cornea. This effect during short-term lens wear mainly occurs because of the thickening of the midperipheral cornea. With long-term lens wear, however, it is associated with not only thickening of the midperipheral cornea but also thinning of the central corneal epithelium [31, 32]. Decreased density in the epithelial basal cells and keratocytes was observed with confocal microscopy in human eyes treated with OK lens wear for 5 years, suggesting that thinning of the central corneal epithelium with long-term OK lens wear may be related to lower metabolic turnover of the entire cornea or increased apoptosis of the keratocytes in the central cornea [32]. However, the CCT remained unchanged in the eyes treated with OK lens wear for 5 years, which was similar to our study results. This may have occurred because of the rearrangement in corneal topography after long-term OK lens wear. To detect these changes after OK treatment, the DCR parameter ARTh has priority over CCT. ARTh indicates the changes in the corneal thickness profile in the temporal-nasal meridian with the following equation: ARTh = corneal thickness thinnest/pachymetric progression [33]. Small ARTh values indicate a rapid increase in corneal thickness toward the periphery and/or a thinner cornea [34]. Our study demonstrated that ARTh decreased significantly after 1 year of OK lens treatment. The multiple linear regression test showed that ARTh was an independent factor associated with both myopia correction and AE. Subjects with smaller ARTh after wearing OK lenses had better myopic correction and slower AE. Based on these results, ARTh may have the potential to be used as an indicator of OK treatment outcomes. Further research to elucidate its role in OK treatment outcomes will help physicians with treatment planning and follow-up.

The mechanism of OK treatment to inhibit AE is still unclear. IOP is a biomechanical factor leading to AE, but neither IOP nor bIOP in this study was associated with AE, in contrast to the results of previous studies [35, 36]. Low stiffness indicates poor ability to resist ocular deformation by increasesd IOP. Previous studies involving ORA showed that lower CH was associated with longer axial length, and that lower CH and CRF may be risk factors for AE [24, 25]; however, the association between the corneal stiffness parameter SP-A1 and AE was not observed during this study. Except for ARTh, our results indicated that older patients exhibited slower AE, which is consistent with the results of previous studies [10, 34, 37]. Because younger children usually experience faster rates of axial elongation, it is difficult to determine whether the association between age and AE is caused by the treatment effect of OK lenses or the natural progression of myopia with age.

After at least 1 year of overnight OK treatment, the residual manifest refraction was -0.64 ± 0.64 D at 4 h after removing the OK lenses, and the distant uncorrected visual acuity was -0.1 ± 0.2 logMAR. The 1-year AE was 0.19 ± 0.12 mm in the OK group and 0.48 ± 0.30 mm in the SVS group. The effect of slowing AE was consistent with the results of previous research [8, 10]; however, the effect of correcting myopia was not as good as that observed during the LORIC study [8]. In this study, there were 13 eyes with a higher myopic degree (>-5.00 D), and the mean SE was -4.97±0.25 D. After excluding the patients with higher degrees of myopia, the mean SE was -4.45±0.31 D and the residual SE was -0.47±0.17 D in our study; however, the respective values were -2.27±1.09 D and -0.18 ± 0.69 D in the LORIC study [8]. The higher baseline SE in our study might explain why the residual SE was larger than that in the LORIC study. Even though residual myopia was corrected with SVS during the daytime, AE in these patients was still smaller than that of the SVS group with the same SE but larger than that of the 33 eyes with <-5.00 D. These results suggested that OK treatment could also be an effective modality to slow AE in patients with higher myopic degrees (>-5.00 D). Patients with higher degrees of myopia and faster AE may try OK treatment plus SVS. However, which patients will benefit from OK treatment requires further study.

The major limitation of the current study was its cross-sectional design. Although there was a control group, the baseline biomechanical properties and their changes are unknown. The results can only indicate some correlated factors. Therefore, the actual role of these factors in the OK treatment outcomes requires further prospective cohort studies.

## Conclusions

In conclusion, long-term OK treatment seems to slow myopia progression by reshaping the cornea. Among these parameters, ARTh was an independent factor associated with both myopic correction and AE. Further prospective studies are required to validate our findings.

## Data Availability

The datasets used and/or analysed during the current study are available from the corresponding author on reasonable request.

## Abbreviations

OK: Orthokeratology
SVS: Single-vision spectacles
AE: Axial elongation
ARTh: Ambrósio’s relational thickness to the horizontal profile
CCT: Central corneal thickness
SE: Spherical equivalent
ORA: Ocular Response Analyzer
CH: Corneal hysteresis
CRF: Corneal resistance factor
DCR: Dynamic corneal response
CBI: Corvis biomechanical idex
SP-A1: Stiffness parameter at first applanation
SP-HC: Highest concavity
IOP: Intraocular pressure
bIOP: Biomechanically corrected IOP
A1: First applanation
A2: Second applanation
HC: Highest concavity
SD: Standard deviation
RGP: Rigid gas-permeable
